# Optimized monoclonal antibody treatment against ELTD1 for GBM in a G55 xenograft mouse model

**DOI:** 10.1111/jcmm.14867

**Published:** 2019-12-21

**Authors:** Michelle Zalles, Nataliya Smith, Jadith Ziegler, Debra Saunders, Shannon Remerowski, Lincy Thomas, Rafal Gulej, Nadya Mamedova, Megan Lerner, Kar‐Ming Fung, Junho Chung, Kyusang Hwang, Junyeong Jin, Graham Wiley, Chase Brown, James Battiste, Jonathan D. Wren, Rheal A. Towner

**Affiliations:** ^1^ Advanced Magnetic Resonance Center Oklahoma Medical Research Foundation Oklahoma City OK USA; ^2^ Oklahoma Center for Neuroscience University of Oklahoma Health Sciences Center Oklahoma City OK USA; ^3^ Department of Pathology University of Oklahoma Health Sciences Center Oklahoma City OK USA; ^4^ Dean McGee Eye Institute University of Oklahoma Health Sciences Center Oklahoma City OK USA; ^5^ Center for Veterinary Sciences Oklahoma State University Stillwater OK USA; ^6^ The Jimmy Everest Center for Cancer and Blood Disorders in Children University of Oklahoma Health Sciences Center Oklahoma City OK USA; ^7^ Pharmaceutical Department Medical University of Lodz Lodz Poland; ^8^ Surgery Research Laboratory University of Oklahoma Health Sciences Center Oklahoma City OK USA; ^9^ Cardiovascular Biology Oklahoma Medical Research Foundation Oklahoma City OK USA; ^10^ Stephenson Cancer Center University of Oklahoma Health Sciences Center Oklahoma City OK USA; ^11^ Department of Biochemistry and Molecular Biology Seoul National University College of Medicine Seoul Korea; ^12^ Clinical Genomics Center Oklahoma Medical Research Foundation Oklahoma City OK USA; ^13^ Genes & Human Disease Oklahoma Medical Research Foundation Oklahoma City OK USA; ^14^ Department of Neurology University of Oklahoma Health Sciences Center Oklahoma City OK USA

**Keywords:** angiogenesis, ELTD1, glioblastoma (GBM), molecular‐targeted MRI, monoclonal antibody (mAb), MRI, notch, orthotopic G55 xenograft model, relative cerebral blood flow (rCBF)

## Abstract

Glioblastoma is an aggressive brain tumour found in adults, and the therapeutic approaches available have not significantly increased patient survival. Recently, we discovered that ELTD1, an angiogenic biomarker, is highly expressed in human gliomas. Polyclonal anti‐ELTD1 treatments were effective in glioma pre‐clinical models, however, pAb binding is potentially promiscuous. Therefore, the aim of this study was to determine the effects of an optimized monoclonal anti‐ELTD1 treatment in G55 xenograft glioma models. MRI was used to assess the effects of the treatments on animal survival, tumour volumes, perfusion rates and binding specificity. Immunohistochemistry and histology were conducted to confirm and characterize microvessel density and Notch1 levels, and to locate the molecular probes. RNA‐sequencing was used to analyse the effects of the mAb treatment. Our monoclonal anti‐ELTD1 treatment significantly increased animal survival, reduced tumour volumes, normalized the vasculature and showed higher binding specificity within the tumour compared with both control‐ and polyclonal‐treated mice. Notch1 positivity staining and RNA‐seq results suggested that ELTD1 has the ability to interact with and interrupt Notch1 signalling. Although little is known about ELTD1, particularly about its ligand and pathways, our data suggest that our monoclonal anti‐ELTD1 antibody is a promising anti‐angiogenic therapeutic in glioblastomas.

## 1 INTRODUCTION

Of all malignant gliomas diagnosed in adults, 82% are characterized as glioblastoma (GBM), which has an incidence of 3.19 per 100 000 persons in the United States.^1^, ^2^ This high‐grade glioma undergoes unregulated vascular angiogenesis and is characterized as being invasive, highly vascular and resistant to apoptosis.^3^ The current treatment plan is surgical resection followed by a combination of radiation and chemotherapy with temozolomide or bevacizumab.^4^ However, even with treatments, the median survival for patients is only 12‐14 months post‐detection, and <5% of patients survive past 5 years post‐diagnosis.^2^, ^4^ GBMs undergo gene amplification and/or mutation of the epidermal growth factor (EGF) receptor and higher EGFR levels were shown to promote migration, tumour growth and angiogenesis.^5^ Gliomas rely heavily on angiogenesis for tumour growth, and the new vessels are the key for delivering oxygen and nutrients to the tumour site. Throughout the years, the primary focus among the pro‐angiogenic factors was the vascular endothelial growth factor (VEGF) for its role of increasing vascularization in cancer. While the tumour develops, there is an up‐regulation of pro‐angiogenic cytokines in the region that further increase VEGF‐A, along with other microvasculature proliferation factors such as basic fibroblast growth factor (bFGF) and epidermal growth factor (EGF).^6^ Once up‐regulated, VEGF‐A binds onto VEGF receptor 2 (VEGFR2) on endothelial cells to initiate a cascade of signalling pathways that promote the formation of new blood vessels.^3^ Bevacizumab is a monoclonal therapy against VEGF‐A approved as a GBM therapeutic agent along with multiple other cancers.^4^ However, this chemotherapeutic agent has not significantly increased the survival of patients suffering with GBM. Furthermore, bevacizumab has serious adverse side effect such as severe/fatal haemorrhaging that occurs up to fivefold more frequently.^7^ Due to Bevacizumab's failure to increase patient's survival, it was crucial to shift the focus from VEGF to other angiogenic factors present in GBMs.

The epidermal growth factor, latrophilin, and seven transmembrane domain‐containing protein on chromosome 1 (ELTD1), alternatively known as the adhesion G protein‐coupled receptor L4 (ADGRL4), was first discovered in developing cardiomyocytes.^8^ ELTD1, a novel regulator of brain angiogenesis, was found to promote tumour growth and metastasis.^9^ We previously reported that ELTD1 was highly expressed in high‐grade gliomas and was expressed on both endothelial and tumour cells.^8^ Furthermore, ELTD1 expression was shown to be regulated by the two main angiogenic pathways, where VEGF increased ELTD1 expression, and DLL4‐Notch signalling decreased ELTD1 expression in normal vasculature.^9^ Further investigation into ELTD1 demonstrated that increased signalling from VEGF‐A resulted in an increase of ELTD1 expression in endothelial cells, and that targeting ELTD1 had decreased VEGFR2 expression in a glioma model.^10^, ^11^

There are approximately 17 000 new GBM diagnoses every year, increasing the need for new and more effective cancer therapeutics.^1^ Our group found that polyclonal antibody (pAb) treatments against ELTD1 in orthotropic GL261 and human G55 xenograft glioma pre‐clinical models were successful in decreasing tumour volumes (TV), increasing survival and decreasing microvessel density levels (MVD) when compared to untreated (UT) control.^12^ However, batch‐to‐batch variabilities as well as potential promiscuity of the pAb posed concerns about specificity as long‐term treatment for patients. Monoclonal antibodies (mAb) are produced from a single B cell clone that allows for homogeneous antibodies and are established as a successful class of targeted treatments for various cancers and chronic inflammatory diseases.^13^ Emerging mAb treatments bind onto growth factors overexpressed on the tumour to disrupt downstream signalling effects to decrease tumour cell growth, proliferation and migration.^14^

Previous research has demonstrated that pAb treatment against ELTD1 was an effective treatment in GBM pre‐clinical models. This study used an optimized monoclonal antibody (mAb) against ELTD1 that has a higher specificity by only binding to the external region of the receptor (430 AA) overcoming the limitations set by the pAb treatments in hopes of obtaining a more specific and profound effect on a G55 glioma pre‐clinical model.

## 2 MATERIALS AND METHODS

### 2.1 Preparation of recombinant extracellular domain of ELTD1 human C_kappa_ fusion protein

To construct extracellular domain of human ELTD1 and mouse ELTD1 expression vectors, genes encoding the human ELTD1 (Glu20‐Leu406) and mouse ELTD1 (Glu20‐Leu455) were chemically synthesized (Genscript, Picataway, NJ, USA). The genes were subcloned into the modified pCEP4 vector encoding C_κ_ domain (human immunoglobulin κ light chain constant domain) at the 5′ region as reported previously.^15^

The expression vectors encoding extracellular domain of human ELTD1 and mouse ELTD1 were transfected into HEK293F cells (Invitrogen, Carlsbad, CA, USA) using 25‐kD linear polyethyleneimine (Polyscience), as reported previously.^16^ Human and mouse ELTD1 C_κ_ fusion proteins were purified from the culture supernatants by affinity chromatography using KappaSelect resin (GE Healthcare) according to the manufacturer's instructions.

### 2.2 Generation of anti‐ELDT1 antibody

White leghorn chickens were immunized with human ELTD1 C_κ_ fusion proteins. A phage‐displayed chicken single‐chain variable fragment (scFv) library was constructed using total RNA isolated from the bone marrow, spleen and bursa of Fabricius of immunized chickens, as described previously.^17^ Positive clones were enriched by biopanning and screened in a phage enzyme immunoassay, as described previously.^18^ Phage clones showing cross reactivity against human and mouse ELTD1 were selected, and their nucleotide sequences were determined by Sanger sequencing. The gene of selected scFv clone was subcloned into a modified mammalian expression vector encoding the hinge region of human IgG1 and the CH2‐CH3 domains of rabbit IgG at the 3′ region as reported previously.^19^ The expression vectors encoding anti‐ELTD1 scFv‐rFc fusion were transfected into HEK293F cells (Invitrogen) as described above. The scFv‐rFc fusion protein was purified from the culture supernatants of transiently transfected HEK293F cells using protein A Sepharose column (Repligen) according to the manufacturer's instructions.

### 2.3 Enzyme immunoassay

96‐well microtiter plate wells (Corning Inc, Corning, NY, USA) were coated with human ELTD1 or mouse ELTD1 C_κ_ fusion protein in coating buffer (0.1M NaHCO_3_, pH 8.6) and then blocked with 3% (w/v) BSA in phosphate‐buffered saline (PBS). After incubation with serially, 10‐fold diluted anti‐ELTD1 scFv‐rFc fusion protein (0.01‐100 nM) and horseradish peroxidase (HRP)‐conjugated goat anti‐rabbit IgG (Fc specific) (Jackson Immuno Research, Inc) were added to each well. After washing with 0.05% (v/v) Tween 20 in PBS (PBST), ABTS HRP substrate solution (Thermo‐Scientific Pierce) was added and the absorbance was measured at 405 nm with a Multiscan Ascent microplate reader (LabSystems).

### 2.4 G55 Xenograft model and treatment

All animal studies were conducted with the approval (protocol 17‐48) of the Oklahoma Medical Research Foundation Institutional Animal Care Use Committee policies, which follow NIH guidelines. Human G55 xenograft cells were implanted intracerebrally in 2‐month‐old male mice (Hsd:Athymic Nude‐Foxn1nu mice; Harlan Inc), as previously described.^10^, ^12^ The animals were divided into three groups: UT, pAb and mAb anti‐ELTD1 treated. Once tumours reached 6‐7 mm^3^ (determined via MRI), mice were either left UT or were treated with 2 mg/kg of either polyclonal anti‐ELTD1 (Bioss, ETL/ELTD1 Polyclonal Antibody, bs‐13111R) or an optimized mAb against ELTD1 every 3‐4 days (treated M/Th, T/F, W/Sat). All mice were euthanized when tumours reached ≥150 mm^3^.

### 2.5 In vivo magnetic resonance (MR) techniques

#### 2.5.1 Morphological imaging

Mice were anesthetized and positioned in a cradle. A 30‐cm horizontal bore Bruker Biospin magnet operating at 7 T (Bruker BioSpin GmbH) was used. A BA6 gradient set and mouse head coil were used to perform all MRI experiments as previously described.^12^ All animals were imaged every 2‐3 days until the end of the study starting at 10 days post‐G55 implantation surgery.

#### 2.5.2 Perfusion imaging

The perfusion imaging method, arterial spin labelling, was used as previously described.^20^ Perfusion maps were obtained on a single axial slice of the brain located on the point of the rostro‐caudal axis where the tumour had the largest cross section. Five regions of interest (ROIs) were manually outlined around the tumour, and appropriate ROIs were also taken from the contralateral side of the brain for comparison purposes. To calculate the differences in (rCBF) values, tumour rCBF values were obtained at late (prior to termination) and early (at tumour detection) tumour stages and normalized to rCBF values in the contralateral brain region of corresponding animals.

#### 2.5.3 Molecular‐targeted MR imaging (mt‐MRI)

The contrast agent, biotin‐BSA (bovine serum albumin)‐Gd (gadolinium)‐DTPA, was prepared as previously described by our group,^12^ based on the modification of the method developed by Dafni et al^21^, ^22^ pAb anti‐ELTD1 (Bioss) or mAb anti‐ELTD, were conjugated to the albumin moiety through a sulfo‐NHS‐EDC link according to the protocol of Hermanson.^23^ mt‐MRI was performed when tumour volumes were around 130‐180 mm^3^. Molecular probes with a biotin‐albumin‐Gd‐DTPA construct bound to anti‐ELTD1 antibodies were injected via a tail vein catheter in mice. A non‐specific mouse immunoglobulin IgG Ab (Alpha Diagnostics) was used with the biotin‐albumin‐Gd‐DTPA construct as a negative control. MRI was done as previously described.^10^, ^21^ Relative probe concentrations were calculated to assess the levels of ELTD1and the non‐specific IgG contrast agent in each animal. Contrast difference images were created from the pre‐ and (90 minutes) post‐contrast datasets for the slice of interest, by computing the difference in T_1_ relaxation times between the post‐contrast and the pre‐contrast image on a pixel basis. From difference images, ten ROIs of equal size (0.05 cm^2^) were drawn within areas with the highest T_1_ relaxation at the TR 800 ms, in the tumour parenchyma and contralateral side of the brains of each animal, after anti‐ETLD1 probe injections. T_1_ values obtained from the ROIs in the tumour regions were normalized to the corresponding contralateral sides. The T_1_ relaxation values of the specified ROIs were computed from all pixels in the ROIs, by the following equation taken (processed by ParaVision 5.0, Bruker): S (TR) = S_0_ (1 − e^−TR/T1^), where TR is the repetition time, S_0_ is the signal intensity (integer machine units) at TR, T_1_ and TE = 0 and T_1_ is the constant of the longitudinal relaxation time.^24^ Overlays of contrast difference images and T_1_‐weighted images were generated using Photoshop software (version C.S 6).

### 2.6 Immunohistochemistry and standard staining

All mice were euthanized after the last MRI examination. The brain of each animal was removed, preserved in 10% neutral buffered formalin, and processed routinely. Haematoxylin‐eosin staining: tissues were fixed in 10% neutral buffered formalin, dehydrated and embedded in paraffin. Sections were deparaffinized, rehydrated and stained according to standard protocols. Several reagents were produced by Vector Labs Inc (VLI).

Histological sections (5 µm) embedded in paraffin and mounted on HistoBond^®^Plus slides (Statlab Medical Products) were rehydrated and washed in phosphate‐buffered saline (PBS). The sections were processed using the ImmPRESS™ VR Reagent Anti‐Rabbit IgG Peroxidase (VLI cat #MP‐6401). Antigen retrieval (pH6 citrate antigen unmasking solution; VLI cat#H‐3300) was accomplished via 20 minutes in a steamer followed by 30 minutes cooling at room temperature. Sections were treated with a peroxidase blocking reagent (Bloxall, VLI cat#SP‐6000), followed by 2.5% normal horse serum to inhibit non‐specific binding. Rabbit Anti‐CD34 antibody (abcam81289; 5.28 µg/mL) or Rabbit Anti‐NOTCH 1 (abcam52627; 11 µg/mL) was applied to each section and following incubation overnight (4°C) in a humidified chamber, sections were washed in PBS, the ImmPRESS VR reagent was applied according to the manufacturer's directions.

To characterize MVD and Notch expression levels, five ROIs, captured digitally (20×), were identified in each case. Only areas containing tumour tissue were analysed, excluding areas with necrosis and/or significant artifacts. The number of positive pixels was divided by the total number of pixels (negative and positive) in the analysed area. ROIs were analysed and imaged using Aperio ImageScope (Leica Biosystems).

Sections for streptavidin horse radish peroxidase (SA‐HRP) were processed as above, except they were incubated overnight with ready to use (RTU) Strp‐HRP (VLI cat#SA‐5704). Appropriate washes were in PBS. Slides were incubated with NovaRed^®^ (VLI cat#SK‐4805) chromogen for visualization. Counterstaining was carried out with Hematoxylin QS Nuclear Counterstain (VLI). Appropriate positive and negative tissue controls were used.

### 2.7 RNA isolation and preparation

Mice were euthanized after the last MRI examination. Brains were removed, snap frozen and stored at −80°C. Total RNA from tumour tissues from all groups was purified with a RNeasy Mini Kit (Qiagen) and quantified by spectrophotometry (Nanodrop).

Concentration of RNA was ascertained, and overall quality of RNA was verified. Sequencing libraries were generated (Lexogen Quantseq FWD library prep kit) according to the manufacturer's protocol. Briefly, the first strand of cDNA was generated using 5′‐tagged poly‐T oligomer primers, and following RNase digestion, the second strand was generated using 5′‐tagged random primers. A subsequent PCR step with additional primers added the complete adapter sequence to the initial 5′ tags, added unique indices for demultiplexing of samples and amplified the library. Final libraries for each sample were assayed (Agilent Tapestation) for appropriate size and quantity. These libraries were pooled in equimolar amounts (fluorometric analyses). Final pools were absolutely quantified using qPCR (Roche LightCycler 480 instrument with Kapa Biosystems Illumina Library Quantification reagents). Sequencing was performed (Illumina Nextseq 500 instrument) with High Output chemistry and 75‐bp single‐ended reads.

### 2.8 Bioinformatics analysis

Paired‐end fastq files were checked for quality using multiQC,^25^ for which the following mean (standard deviation) descriptive values were 54 million reads (12), 68.8% (2.8%) duplicate reads and a GC content of 51.3% (0.7%). The indexing and alignment were run with kallisto^26^ against build 38 of the human reference genome from the Genome Reference Consortium (GRCh38). Assignment of counts to exon features and normalization were performed along with the alignment via the biojupies pipeline to provide a counts matrix. Significant differential genes were determined by DESeq2^27^ for genes having both a Benjamini‐Hochberg adjusted *P*‐value < .05 and an absolute log fold change of >1.3. Gene set enrichment analysis was performed on the identified differential genes via enrichR^28^ (gseapy API).

### 2.9 Statistical analysis

Survival curves were analysed using Kaplan‐Meier curves. Tumour volumes, perfusion changes, and immunohistochemistry protein levels, and molecular‐targeted MRI data were analysed and compared by one‐ or two‐way ANOVA with multiple comparisons (Tukey's or Sidak's, respectively). Data were represented as mean ± SD, and *P*‐values of either *<.05, **<.01, ***<.001, ****<.0001 were considered statistically significant.

## 3 RESULTS

**Figure 1:**
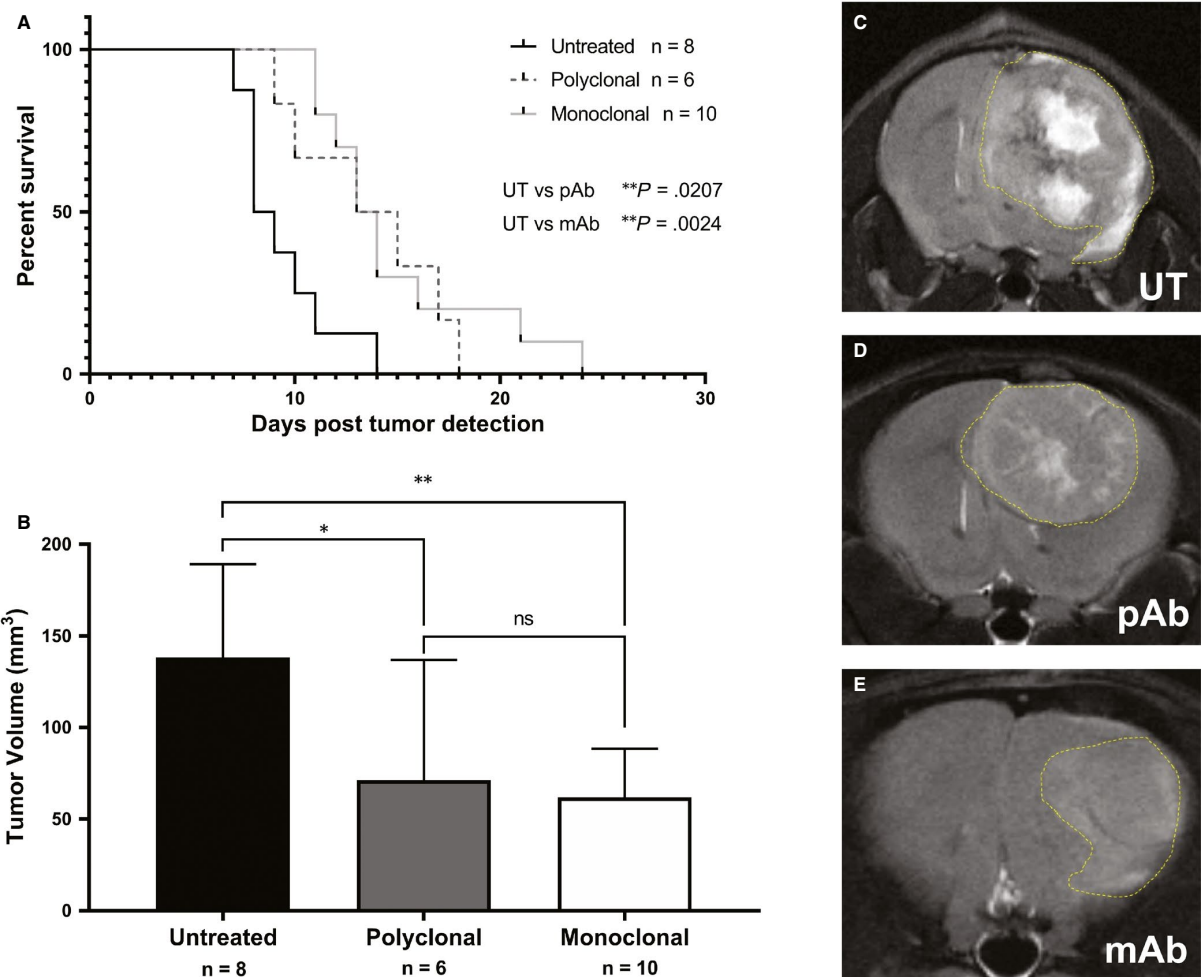
Monoclonal anti‐ELTD1 treatment is more effective in increasing animal survival and decreasing tumour volumes (TV). A, Per cent survival curve for all treatment groups; untreated control. pAb and mAb treatments were able to significantly increase the overall survival post‐tumour detection. B, Tumour volumes of each treatment group 9 days post‐tumour detection. pAb and mAb treatment significantly decreased TV compared with UT (*P = .0384, **P = .0067). Representative morphological MR images for untreated (C), pAb treatment (D) and mAb treatment (E) 9 d post‐tumour detection with the tumour outlined in yellow

Prior studies demonstrated that non‐specific IgG antibody treatments as a control group did not differ from untreated; therefore, in this study, we only utilized untreated animals as the control group.^12^ G55 glioma‐bearing mice treated against ELTD1, both pAb (*P* = .0207) and mAb (*P* = .0024), significantly increased the survival compared with untreated (average survival ~ 9 days post‐tumour detection) as shown in Figure 1A. TVs 9 days post‐tumour detection, monitored via MRI, were significantly lower with the monoclonal (*P* = .0067) and polyclonal (*P* = .0384) anti‐ELTD1 treatment compared with controls (Figure 1B). Representative images of tumour‐bearing mice from all treatment groups are shown in Figure 1C.

**Figure 2:**
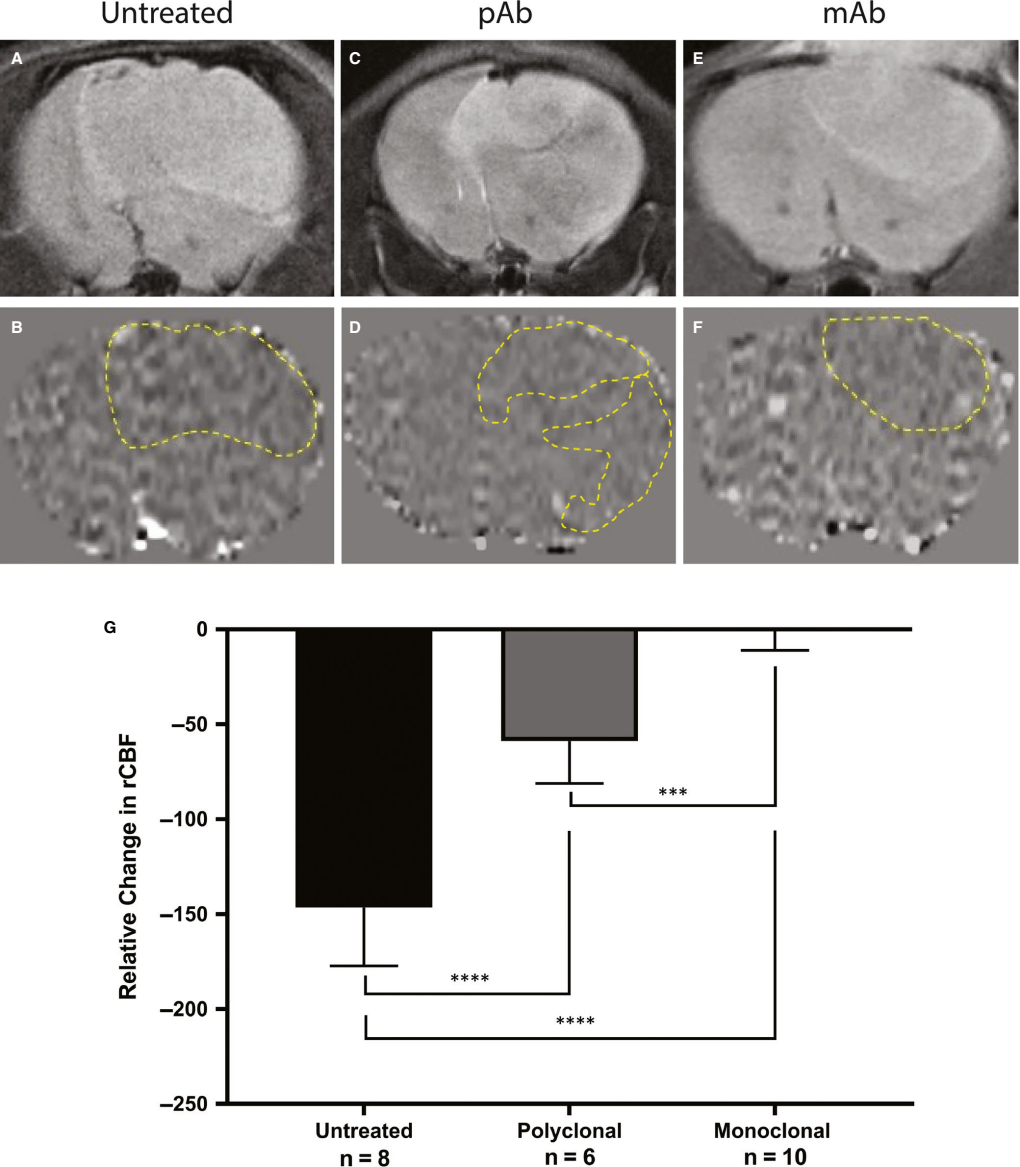
Monoclonal anti‐ELTD1 treatment normalizes vasculature within the tumour. Representative morphological images with respective MR perfusion maps for each treatment group at tumour maximum volume (TV:120‐160 mm^2^): untreated control (A,B), pAb‐treated animals (C,D) and mAb‐treated animals (E,F). G, Quantitative analysis of tumour rCBF differences. The rCBF perfusion levels were significantly increased with both anti‐ELTD1 treatments. The mAb treatment was also able to normalize the perfusion levels (***P = .0001 UT vs pAb, ****P < .0001 UT vs mAb)

MRI perfusion measures the relative cerebral blood flow (rCBF) and can be used to assess the microvasculature alterations associated with tumour angiogenesis. Healthy normal tissue has a set rCBF; however, as the tumour grows, it disrupts the vasculature, and therefore decreases the perfusion rate. Differences in rCBF demonstrated that the untreated mice had a decrease in rCBF in the tumour regions depicting increased angiogenesis while the anti‐ELTD1‐treated animals had a normalization of perfusion values. Representative morphological MRIs along with the corresponding perfusion maps of the brain are depicted in Figure 2A‐F. The decrease in perfusion (depicted as decreased normalized rCBF) as a result of the tumour, outlined by the yellow‐dashed line in 2B, 2D, 2F, is demonstrated by the dark areas. Our monoclonal and polyclonal treatments were successful in decreasing angiogenesis and therefore increasing perfusion within the tumour region. The polyclonal anti‐ELTD1 treatment was able to minimize the decrease in rCBF (*P* < .0001) compared with UT mice. The mAb treatment was significantly more effective in decreasing the rCBF compared with both pAb treatment (*P* = .0001) and UT (*P* < .0001) (Figure 2G) and normalized the rCBF within the tumour region to contralateral levels.

**Figure 3:**
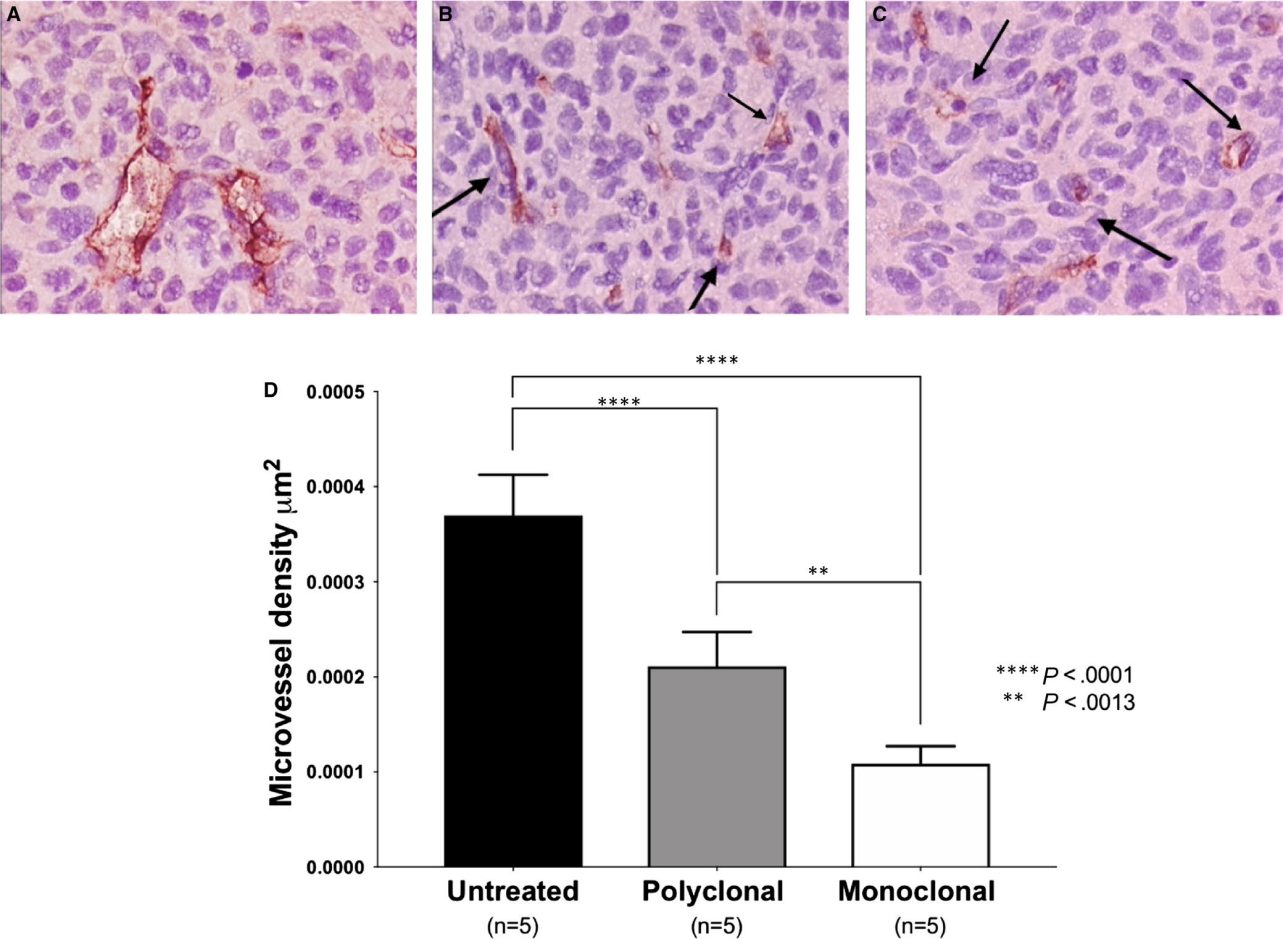
Anti‐ELTD1 antibody therapy is effective in decreasing the microvessel density (MVD). Representative IHC images (20×) for CD34 from untreated (A), polyclonal anti‐ELTD1 (pAb)‐treated (B) and monoclonal anti‐ETLD1 (mAb)‐treated animals (C) at tumour maximum volume (TV 120‐160 mm^2^). Dark red/brown staining in the slides represents vessels in the tumour region highlighted by the arrows. D, MVD analysis for all of the treatment groups. The pAb and mAb treatments were able to significantly decrease MVD (****P < .0001 for both). There was also a significant decrease in MVD for the mAb vs the pAb (**P < .01)

ELTD1 has been linked with pathological angiogenesis. Therefore, we analysed MVD to determine whether the anti‐ELTD1 treatments would alter the tumour vasculature. Representative CD34 IHC images for each treatment groups are shown in Figure 3A‐C. CD34 analysis demonstrated that the anti‐ELTD1 treatments significantly decreased the MVD levels (*P* < .0001) compared with control (Figure 3D). The monoclonal anti‐ELTD1 treatment further decreased the MVD levels compared with the polyclonal anti‐ELTD1 treatment (*P* = .0013) and was able to return the MVD to near normal levels.

**Figure 4:**
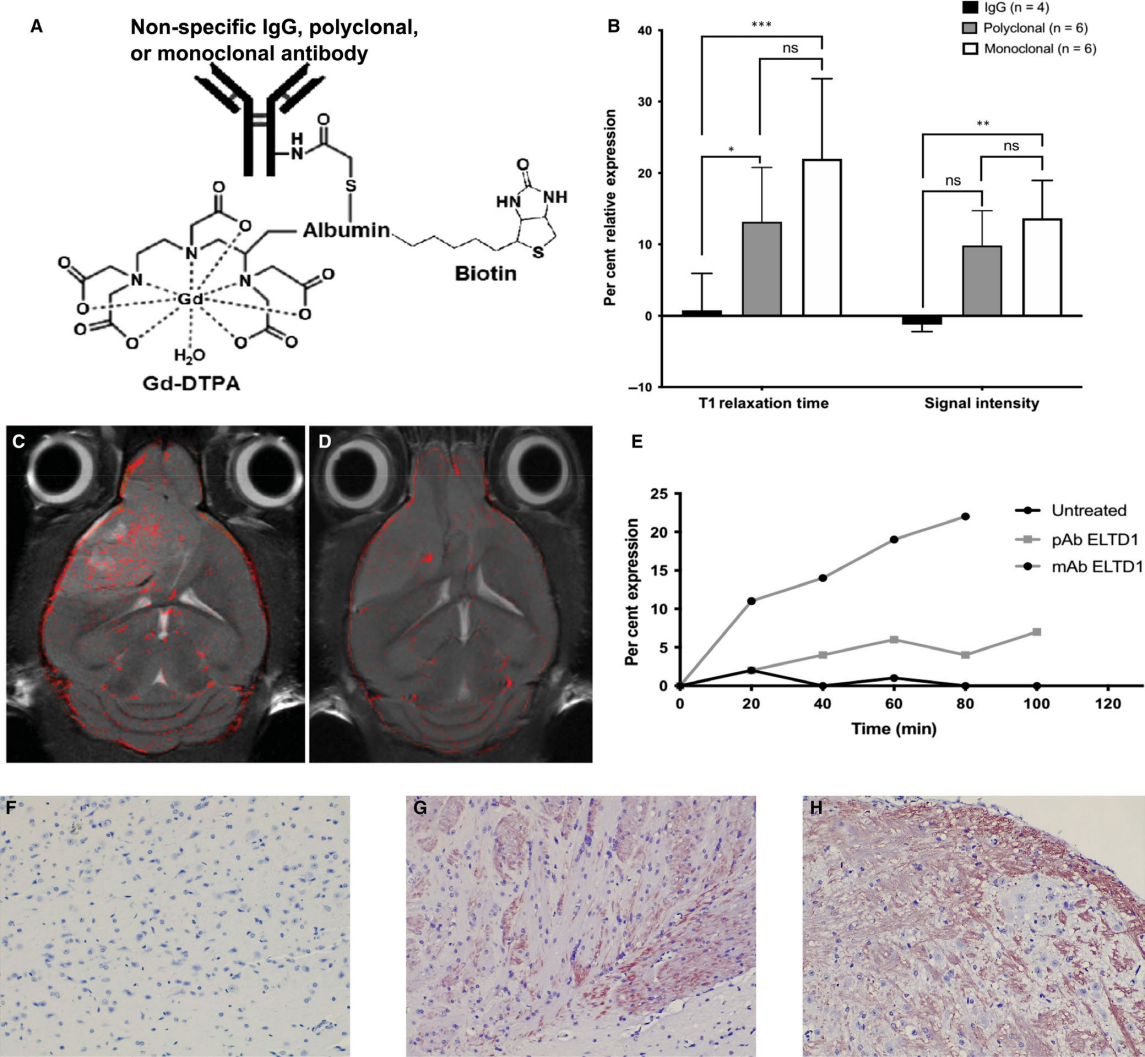
mAb ELTD1 probe has significantly higher binding specificity against the tumour. A, Molecular probe construct. Gd‐DTPA signal was used to detect the probe via MR imaging while the biotin tag allowed for localization in the tumour tissue post‐termination. B, Per cent relative expression of our molecular probes indicates a change in either T1 Relaxation or SI due to the presence of the Gd‐DTPA component. The mAb‐attached probe had significantly higher signal intensity and T1 relaxation time than the IgG control (T1: *P = .0307 (IgG vs pAb ELTD1 probe), ***P = .0002 (IgG vs mAb ELTD1 probe); SI: **P = .008 (IgG vs mAb ELTD1 probe)), (C, D) localization and clustering of our monoclonal‐attached molecular probes (C) and non‐specific IgG‐attached molecular probe (D). E, Kinetics of the antibody‐attached probes, non‐specific IgG control, pAb and mAb against ELTD1. F‐H, Representative images (20×) stained with SA‐HRP to localize the non‐specific IgG‐attached probes (F), pAb‐attached probe (G) and mAb‐attached probe (H) at tumour maximum (TV: 120‐160 mm^2^). The brown staining seen in the pAb‐ and mAb‐attached probes is the localized probes

To determine where our Ab was localizing in vivo, we synthesized a biotin‐BSA (bovine serum albumin)‐Gd‐DTPA probe attached to either non‐specific IgG, polyclonal anti‐ELTD1 or monoclonal anti‐ELTD1 antibodies (Figure 4A). The molecular probes were injected via tail vein catheter into untreated G55‐glioma‐bearing mice, T1 relaxation times and signal intensity were calculated via MRI. T1 relaxation is an MRI contrast parameter that is reduced in the presence of our molecular probe. The results shown in Figure 4B demonstrate the presence of our molecular probe as the per cent relative expression, due to its effect on T1 relaxation. Both T1 (*P* = .0002) and signal intensity (*P* = .008) were significantly increased by the monoclonal anti‐ELTD1‐attached probe compared with the non‐specific IgG‐attached probe. Our polyclonal anti‐ELTD1‐attached probe significantly increased the T1 relaxation (*P* = .0307) but did not significantly affect the signal intensity (*P* = .0602).

Molecular‐targeted MRIs of representative monoclonal anti‐ELTD1 and non‐specific IgG probe data were overlaid onto morphological images of untreated G55 tumour‐bearing animals. Figure 4C demonstrates that the monoclonal anti‐ELTD1‐attached probe had an increased binding specificity against the tumour region. However, the non‐specific IgG probe mainly clustered around the blood vessels (Figure 4D). After monitoring the expression of the molecular probes, we saw that the mAb‐attached probe had a more profound and sustained effect when compared to both the non‐specific IgG and polyclonal anti‐ELTD1‐attached probe (Figure 4E).

Our molecular probe had an attached biotin tag to further localize it in the tissue. Once molecular targeting was concluded, the animal was terminated and their tissue was taken for histology. By staining the tumour tissue with SA‐HRP, we confirmed our molecular targeting results. Our polyclonal‐ and monoclonal‐attached probes were localized in the tumour tissue post‐termination while there were no traces of our non‐specific IgG‐attached probes in the tissue. (Figure 4F‐H). These data demonstrate that the mAb probe has significantly higher binding specificity against the tumour region than both the polyclonal‐attached probe and IgG probe.

**Figure 5:**
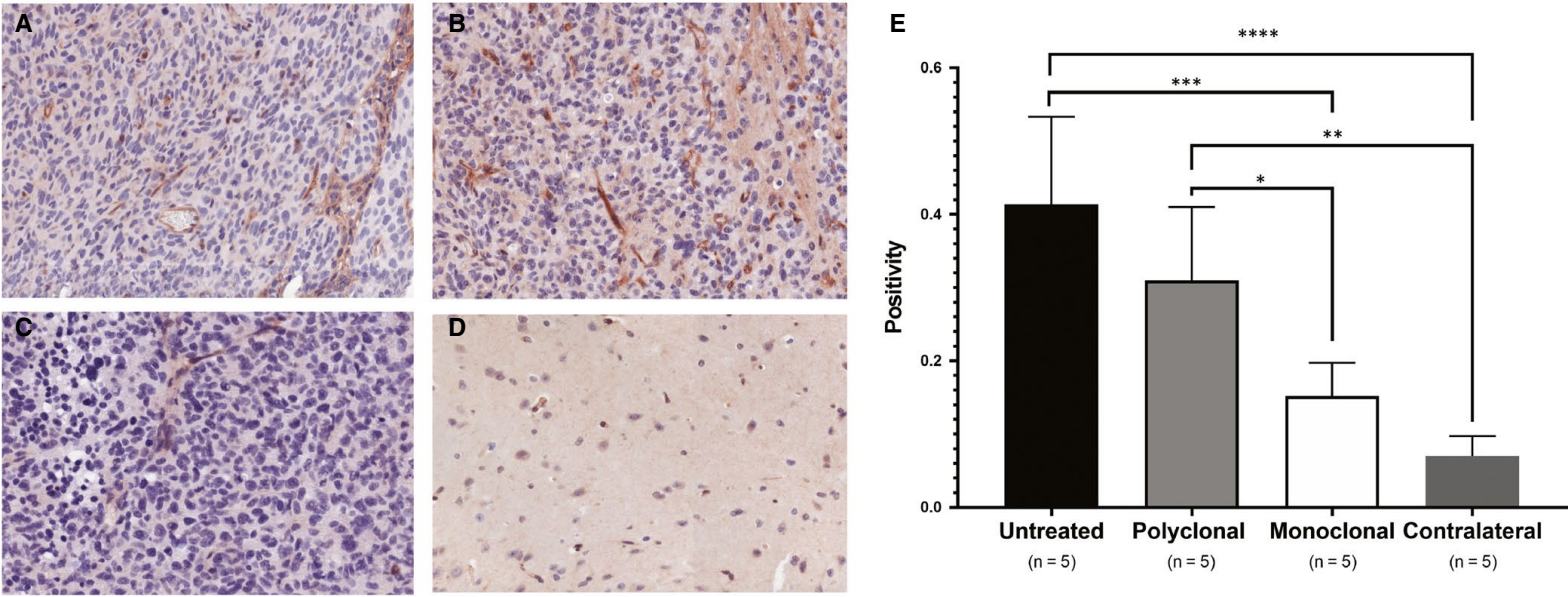
mAb treatment against ELTD1 decrease Notch1 levels. A‐D, Representative images (20×) of IHC stained tumours with Notch1 of untreated (A), pAb treatment (B), mAb treated (C) and contralateral control (D). E, Quantitative positivity Notch staining of the samples. mAb against ELTD1‐treated mice significantly lowered Notch levels when compared to both untreated and pAb‐treated animals. There was no significant difference between untreated *vs* pAb treatment, and mAb treatment and contralateral (healthy control). Contralateral (Cont) tissue Notch levels were significantly lower than untreated mice and pAb‐treated animals (*P = .0357 (mAb vs pAb), **P = .0015 (Cont vs pAb), ***P = .0006 (UT vs mAb), ****P < .0001 (UT vs Cont))

Notch signalling is important for cell differentiation, proliferation, as well as tumour angiogenesis, and normal vasculature has been shown to decrease ELTD1 expression.^29^ Therefore, we examined whether Notch levels changed with anti‐ELTD1 treatment. Positivity analysis of the stained samples demonstrated that the untreated glioma tumour samples had the highest amount of Notch1. Our monoclonal anti‐ELTD1 treatment significantly decreased Notch1 expression levels compared with both pAb treatments (*P* = .0357) and untreated control (*P* = .0006) and brought down the expression to contralateral levels (Figure 5E).

**Figure 6:**
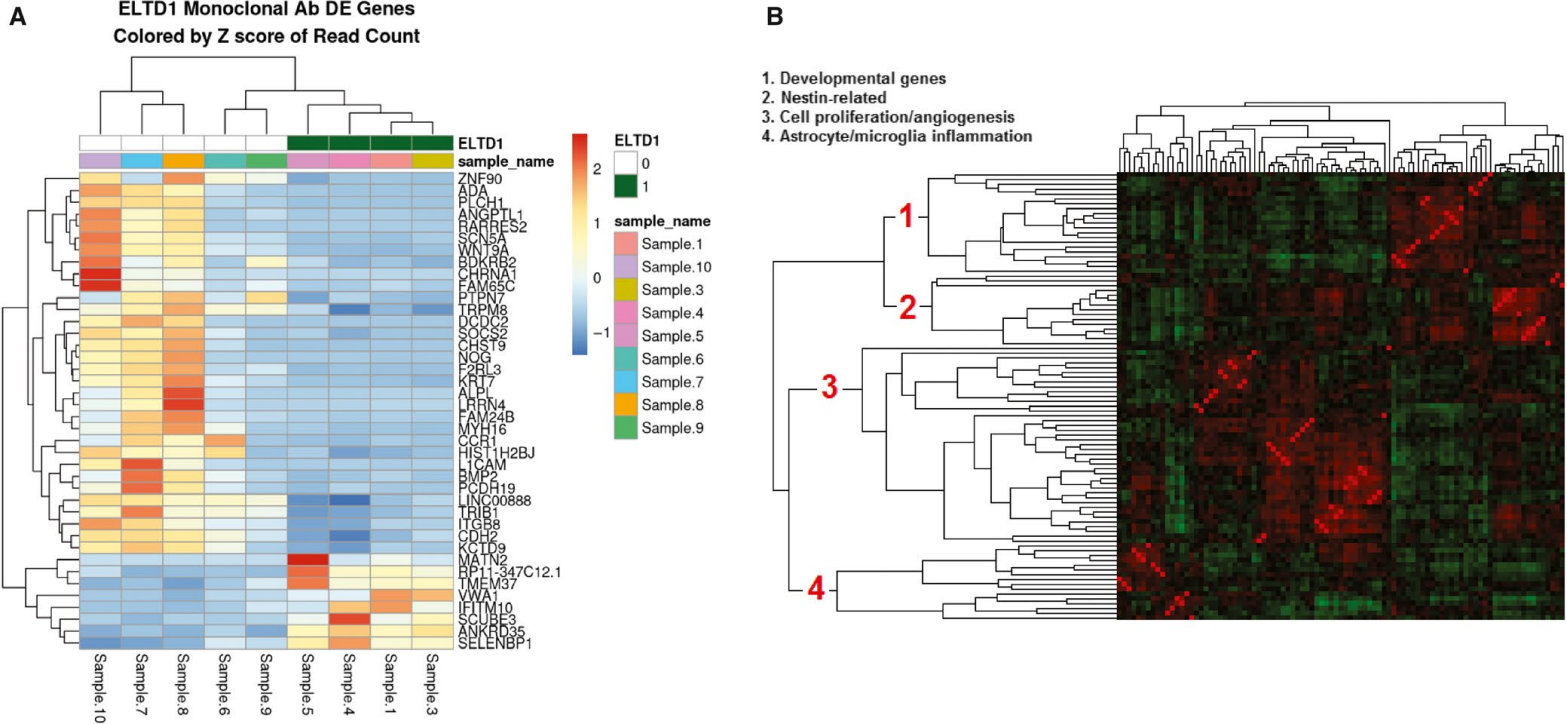
A, Gene‐fold changes when comparing ELTD1 mAb‐treated mice to UT from up‐regulated (red) to down‐regulated (blue), obtained from RNA‐seq analysis. B, Gene‐gene correlations for the genes repressed after anti‐ELT1 mAb treatment. Red = positively correlated, green = negatively correlated. Using literature analysis software^60^ to categorize the groups of genes in terms of their published commonalities, they roughly fall into four categories (developmental genes, nestin‐related, cell proliferation/angiogenesis, astrocyte microglia inflammation)

Our in vivo data demonstrated that the mAb treatment against ELTD1 was more effective in the G55 xenograft model; therefore, we only examined the effect that our mAb treatment (compared to untreated) had on the genes in the tumour region. From all of the genes found in Figure 6A, ADA, SCN5A, L1CAM, BMP2, ALPL, TRPM8, SELENBP1 have been directly associated with gliomas. While other genes were associated with various other cancers such as hepatocellular carcinoma (VWA1^30^), lung cancer (SCUBE3,^31^ PLCH1,^32^ CHRNA1,^33^ CDH2^34^) and breast cancer (IFITM10,^35^ DCDC2,^36^ CHST9,^37^ CDH2^38^). To see whether some of the genes down‐regulated upon anti‐ELTD1 Ab treatment had been similarly co‐regulated in other experiments, we first calculated gene‐gene Pearson's correlations using experiments from the microarray platform GPL570, which are publicly available as part of NCBI's GEO database. Figure 6B shows the clustered gene‐gene correlations of our down‐regulated genes using the GPL570 data. Roughly, 4 clusters (developmental genes, nestin‐related, cell proliferation/angiogenesis, astrocyte microglia inflammation) are apparent, indicating that groups of genes seen as differentially expressed in our experiment have also been observed in other experiments.

## 4 DISCUSSION

Through a global microarray meta‐analysis (GAMMA),^39^ we identified ELTD1, an angiogenic marker, to be highly expressed in high‐grade gliomas and other groups have suggested that high ELTD1 expression levels may correlate with the aggressiveness of the glioma.^29^, ^40^ Previous studies have demonstrated that anti‐ELTD1 treatments with pAb were effective in mouse GL261 and human G55 xenograft glioma models.^12^ Other groups have also discovered that microRNA‐139‐5p directly binds onto and targets ELTD1 to inhibit cell proliferation in gliomas.^41^

This study focuses on an optimized mAb therapy against ELTD1 in a human G55 xenograft glioma mouse model. G55 is a stable xenograft cell line that was initially taken from a human GBM and passaged through nude mice.^42^, ^43^ Historically, this cell line has many characteristics of primary human GBM such as hypervascularity and necrosis and has been used by numerous studies focusing on invasive intracranial tumours.^42^, ^43^, ^44^, ^45^ Our data have shown that repetitive IV treatments with both pAb and mAb against ELTD1 led to a significant decrease in tumour volumes and increase in survival. Prior published work from our laboratory showed a survival increase of 7‐10 days with the pAb ELTD1 treatment; however, this current study only showed an average increase of 5 days.^12^ The discrepancy between studies is due to the different doubling times between our G55 cells. The 2017 study used high‐passaged G55 cells in which the untreated mice had a doubling time of 2.5 days with an average survival of 18 days; however, this current study used low‐passaged G55 cells that appeared more aggressive due to their faster doubling period of 2 days and an average survival of 10 days. The mAb treatment against ELTD1, however, was able to increase the doubling time to approximately 2.7 days even in this more aggressive glioma model.

Our optimized mAb treatment was shown to not only return the perfusion levels, depicting a normalization of the vasculature within the tumour region, but was also effective in significantly decreasing the MVD levels. Together, these data demonstrate that our mAb therapy against ELTD1 has a more profound effect on the tumour‐related microvasculature when compared to both untreated and pAb‐treated animals. Prior studies using human G55 cells have shown increased haemorrhaging using anti‐VEGF therapy in mice, and patients undergoing bevacizumab treatment have shown an increased risk of intracranial haemorrhaging.^7^, ^12^ We did not see any haemorrhaging in our pAb‐ nor mAb‐treated tumour regions on our MR images nor when stained with Prussian blue. Therefore, this suggests that anti‐ELTD1 treatment could potentially be safer for use in the clinic.

Together, our data demonstrated that ELTD1 is an important angiogenic marker in high‐grade gliomas. By using an optimized mAb treatment against ELTD1, we were able to significantly increase survival, decrease TV and normalize the tumour‐associated vasculature. This study demonstrated that our optimized mAb against ELTD1 had higher binding specificity when compared to a commercially used pAb confirmed through molecular targeting and histology.

Prior studies have stated that VEGF increases ELTD1 expression and DLL4‐Notch decreases the expression in normal vasculature; however, our study has demonstrated that this relationship may be more complex in the tumour environment.^9^ We were also able to significantly reduce Notch1 levels within the tumour and return them to near contralateral levels with our mAb treatment. Furthermore, our RNA‐sequencing data demonstrated that 3 of the genes (SCN5A, L1CAM, BMP2) affected with the anti‐ELTD1 treatment that were directly associated with gliomas influenced and interacted with Notch signalling. Therefore, suggesting that ELTD1 has a more complex relationship with Notch1 than previously understood. However, the RNA‐sequence analysis did not show specific gene changes with Notch.

Aside from the possible relationship with Notch, the RNA‐seq data gave further insight as to what pathways the anti‐ELTD1 treatment is targeting. ADA and BMP2 expression are correlated with poor prognosis in glioma patients,^46^, ^47^ which were both down‐regulated with the anti‐ETLD1 treatments. Furthermore, the anti‐ELTD1 treatment worked to down‐regulate SCN5A, TRPM8 and BMP2, which were all shown to increase glioma cell proliferation, migration and invasion.^48^, ^49^, ^50^ Alkaline phosphatase (ALPL) is a stem cell marker, that is highly expressed around necrotic areas within the tumour, and high expression of ALPL and CD133 (another stem cell marker) has been associated with poor prognosis for patients.^51^ The mAb therapy against ELTD1 was successful in down‐regulating ALPL. CD133+ glioma cells show neurosphere‐like growth, drive tumour formation and are resistant to standard therapies.^52^ L1CAM, a gene down‐regulated by anti‐ELTD1 treatment, is overexpressed in GBMs and CD133+ glioma cells and regulates neural cell growth, migration and survival during development.^53^, ^54^ Furthermore, targeting and inhibiting L1CAM in CD133+ glioma cells suppressed tumour growth and increased the survival in a glioma xenograft model.^54^ Glioma stem cells (GSC) are the main cause of GBM recurrence after therapy and are characterized by CD133.^55^ However, recent reports have shown that CD133 may not be a robust marker for GSC and CD133 cells that possess GSC properties may give rise to aggressive tumours.^55^, ^56^ Nestin was first characterized as a neuronal stem cell marker and found on the surface of both CD133‐positive and ‐negative cells and may serve as a more efficent GSC marker in GBMs.^55^, ^56^ Furthermore, nestin has been shown to be a key player in proliferation, migration and survival of GBMs and other cancers.^57^, ^58^, ^59^ Interestingly, cluster gene‐gene analysis from the genes down‐regulated by the anti‐ELTD1 treatment suggests that there is a down‐regulation response for nestin‐related pathways.

Our data demonstrate that an optimized mAb therapy against ELTD1 is a potential anti‐angiogenic therapy against GBMs. Although discovered in 2001, there are various unknowns about ELTD1, including its mechanism of action and its ligand. However, we hope that through our newly obtained RNA‐sequence data, possible ELTD1 pathways of interaction can be examined. Future studies will further focus on ELTD1's relationship with Notch as well as possible ligands of ELTD1.

## CONFLICT OF INTEREST

None of the authors have any conflict of interest.

## AUTHOR CONTRIBUTIONS

MZ performed the research and wrote the manuscript. The manuscript was edited by RT, NS, DS, JW, JC and RG. JZ, DS, NM and SR assisted during the animal surgery. MR imaging and treatment were conducted by MZ, JZ, NS, NM, DS, SR, LT and RG. Molecular‐targeted MR imaging and RNA isolation were conducted by NS and MZ. JC, KH and JJ generated the anti‐ELTD1 monoclonal antibody. MZ, ML, KMF and JC performed and assisted with the IHC and analysis. JW and CB performed RNA‐seq and bioinformatics analyzes. MZ and SR analysed the MRI data. RT was the principle investigator that conceived the study design, oversaw the entire study and performed the data interpretation.

## Data Availability

Data will be made available upon reasonable request.

## References

[1] Dolecek TA , Propp JM , Stroup NE , Kruchko C . CBTRUS statistical report: primary brain and central nervous system tumors diagnosed in the United States in 2005–2009. Neuro Oncol. 2012;14(Suppl 5):v1‐v49. 23095881 10.1093/neuonc/nos218 PMC3480240

[2] Tamimi AF , Juweid M . Epidemiology and outcome of glioblastoma In: De Vleeschouwer S ed. Glioblastoma, Chapter 8. Brisbane, QLD: Codon Publications;2017:143‐144. 29251870

[3] Alves TR , Lima FR , Kahn SA , et al. Glioblastoma cells: a heterogeneous and fatal tumor interacting with the parenchyma. Life Sci. 2011;89:532‐539. 21641917 10.1016/j.lfs.2011.04.022

[4] Beal K , Abrey LE , Gutin PH . Antiangiogenic agents in the treatment of recurrent or newly diagnosed glioblastoma: analysis of single‐agent and combined modality approaches. Radiat Oncol. 2011;6:2. 21214925 10.1186/1748-717X-6-2 PMC3025871

[5] Furnari FB , Fenton T , Bachoo RM , et al. Malignant astrocytic glioma: genetics, biology, and paths to treatment. Genes Dev. 2007;21:2683‐2710. 17974913 10.1101/gad.1596707

[6] Suhardja A , Hoffman H . Role of growth factors and their receptors in proliferation of microvascular endothelial cells. Microsc Res Tech. 2003;60:70‐75. 12500263 10.1002/jemt.10245

[7] Genentech I . Avastin prescribing information; 2018.

[8] Nechiporuk T , Urness LD , Keating MTETL . ETL, a novel seven‐transmembrane receptor that is developmentally regulated in the heart. ETL is a member of the secretin family and belongs to the epidermal growth factor‐seven‐transmembrane subfamily. J Biol Chem. 2001;276:4150‐4157. 11050079 10.1074/jbc.M004814200

[9] Masiero M , Simoes FC , Han HD , et al. A core human primary tumor angiogenesis signature identifies the endothelial orphan receptor ELTD1 as a key regulator of angiogenesis. Cancer Cell. 2013;24:229‐241. 23871637 10.1016/j.ccr.2013.06.004 PMC3743050

[10] Ziegler J , Zalles M , Smith N , et al. Targeting ELTD1, an angiogenesis marker for glioblastoma (GBM), also affects VEGFR2: molecular‐targeted MRI assessment. Am J Nucl Med Mol Imaging. 2019;9:93‐109. 30911439 PMC6420708

[11] Dieterich LC , Mellberg S , Langenkamp E , et al. Transcriptional profiling of human glioblastoma vessels indicates a key role of VEGF‐A and TGFbeta2 in vascular abnormalization. J Pathol. 2012;228:378‐390. 22786655 10.1002/path.4072

[12] Ziegler J , Pody R , Coutinho de Souza P , et al. ELTD1, an effective anti‐angiogenic target for gliomas: preclinical assessment in mouse GL261 and human G55 xenograft glioma models. Neuro Oncol. 2017;19:175‐185. 27416955 10.1093/neuonc/now147 PMC5464087

[13] Buss NA , Henderson SJ , McFarlane M , Shenton JM , de Haan L . Monoclonal antibody therapeutics: history and future. Curr Opin Pharmacol. 2012;12:615‐622. 22920732 10.1016/j.coph.2012.08.001

[14] Reichert JM , Rosensweig CJ , Faden LB , Dewitz MC . Monoclonal antibody successes in the clinic. Nat Biotechnol. 2005;23:1073‐1078. 16151394 10.1038/nbt0905-1073

[15] Lee Y , Kim H , Chung J . An antibody reactive to the Gly63‐Lys68 epitope of NT‐proBNP exhibits O‐glycosylation‐independent binding. Exp Mol Med. 2014;46:e114. 25236766 10.1038/emm.2014.57 PMC4183943

[16] Boussif O , Lezoualc'h F , Zanta MA , et al. A versatile vector for gene and oligonucleotide transfer into cells in culture and in vivo: polyethylenimine. Proc Natl Acad Sci USA. 1995;92:7297‐7301. 7638184 10.1073/pnas.92.16.7297 PMC41326

[17] Andris‐Widhopf J , Rader C , Steinberger P , Fuller R , Barbas CF 3rd . Methods for the generation of chicken monoclonal antibody fragments by phage display. J Immunol Methods. 2000;242:159‐181. 10986398 10.1016/s0022-1759(00)00221-0

[18] Barbas CF . Phage display: a laboratory manual. Cold Spring Harbor, NY: Cold Spring Harbor Laboratory Press; 2001.

[19] Han J , Lee JH , Park S , et al. A phosphorylation pattern‐recognizing antibody specifically reacts to RNA polymerase II bound to exons. Exp Mol Med. 2016;48:e271. 27857068 10.1038/emm.2016.101 PMC5133369

[20] Zhu W , Kato Y , Artemov D . Heterogeneity of tumor vasculature and antiangiogenic intervention: insights from MR angiography and DCE‐MRI. PLoS ONE. 2014;9:e86583. 24466160 10.1371/journal.pone.0086583 PMC3900564

[21] Towner RA , Smith N , Doblas S , et al. In vivo detection of inducible nitric oxide synthase in rodent gliomas. Free Radic Biol Med. 2010;48:691‐703. 20034558 10.1016/j.freeradbiomed.2009.12.012

[22] Dafni H , Landsman L , Schechter B , Kohen F , Neeman M . MRI and fluorescence microscopy of the acute vascular response to VEGF165: vasodilation, hyper‐permeability and lymphatic uptake, followed by rapid inactivation of the growth factor. NMR Biomed. 2002;15:120‐131. 11870908 10.1002/nbm.724

[23] Hermanson G . Bioconjugate techniques. New York, NY: Academic Press; 1996.

[24] Haacke E . Magnetic resonance imaging: physical principles and sequence design. New York, NY: Wiley‐Liss; 1999.

[25] Ewels P , Magnusson M , Lundin S , Kaller M . MultiQC: summarize analysis results for multiple tools and samples in a single report. Bioinformatics. 2016;32:3047‐3048. 27312411 10.1093/bioinformatics/btw354 PMC5039924

[26] Torre D , Lachmann A , Ma'ayan A . BioJupies: automated generation of interactive notebooks for RNA‐seq data analysis in the cloud. Cell Syst. 2018;7(556–61):e3. 10.1016/j.cels.2018.10.007 PMC6265050 30447998

[27] Love MI , Huber W , Anders S . Moderated estimation of fold change and dispersion for RNA‐seq data with DESeq2. Genome Biol. 2014;15:550. 25516281 10.1186/s13059-014-0550-8 PMC4302049

[28] Chen EY , Tan CM , Kou Y , et al. Enrichr: interactive and collaborative HTML5 gene list enrichment analysis tool. BMC Bioinf. 2013;14:128. 10.1186/1471-2105-14-128 PMC3637064 23586463

[29] Serban F , Daianu O , Tataranu LG , et al. Silencing of epidermal growth factor, latrophilin and seven transmembrane domain‐containing protein 1 (ELTD1) via siRNA‐induced cell death in glioblastoma. J Immunoassay Immunochem. 2017;38:21‐33. 27379831 10.1080/15321819.2016.1209217

[30] Zou Q , Xiao Z , Huang R , et al. Survey of the translation shifts in hepatocellular carcinoma with ribosome profiling. Theranostics. 2019;9:4141‐4155. 31281537 10.7150/thno.35033 PMC6592166

[31] Zhao C , Qin Q , Wang Q , et al. SCUBE3 overexpression predicts poor prognosis in non‐small cell lung cancer. Biosci Trends. 2013;7:264‐269. 24390364

[32] Zhang Y , Hua S , Zhang A , et al. Association between polymorphisms in COMT, PLCH1, and CYP17A1, and non‐small‐cell lung cancer risk in Chinese nonsmokers. Clin Lung Cancer. 2013;14:45‐49. 22658813 10.1016/j.cllc.2012.04.004

[33] Chang PM , Yeh YC , Chen TC , et al. High expression of CHRNA1 is associated with reduced survival in early stage lung adenocarcinoma after complete resection. Ann Surg Oncol. 2013;20:3648‐3654. 23775407 10.1245/s10434-013-3034-2

[34] Zhuo H , Zhao Y , Cheng X , et al. Tumor endothelial cell‐derived cadherin‐2 promotes angiogenesis and has prognostic significance for lung adenocarcinoma. Mol Cancer. 2019;18:34. 30832661 10.1186/s12943-019-0987-1 PMC6399986

[35] Varley KE , Gertz J , Roberts BS , et al. Recurrent read‐through fusion transcripts in breast cancer. Breast Cancer Res Treat. 2014;146:287‐297. 24929677 10.1007/s10549-014-3019-2 PMC4085473

[36] Cai Y , Li WF , Sun Y , Liu K . Downregulation of microRNA‐645 suppresses breast cancer cell metastasis via targeting DCDC2. Eur Rev Med Pharmacol Sci. 2017;21:4129‐4136. 29028086

[37] Yuan J , Zhang N , Zhu H , et al. CHST9 rs1436904 genetic variant contributes to prognosis of triple‐negative breast cancer. Sci Rep. 2017;7:11802. 28924212 10.1038/s41598-017-12306-6 PMC5603563

[38] Lee JW , Guan W , Han S , Hong DK , Kim LS , Kim H . MicroRNA‐708‐3p mediates metastasis and chemoresistance through inhibition of epithelial‐to‐mesenchymal transition in breast cancer. Cancer Sci. 2018;109:1404‐1413. 29575368 10.1111/cas.13588 PMC5980212

[39] Wren JD . A global meta‐analysis of microarray expression data to predict unknown gene functions and estimate the literature‐data divide. Bioinformatics. 2009;25:1694‐1701. 19447786 10.1093/bioinformatics/btp290 PMC2732319

[40] Towner RA , Jensen RL , Colman H , et al. ELTD1, a potential new biomarker for gliomas. Neurosurgery. 2013;72:77‐90; discussion 1. 10.1227/NEU.0b013e318276b29d PMC3555701 23096411

[41] Dai S , Wang X , Li X , Cao Y . MicroRNA‐139‐5p acts as a tumor suppressor by targeting ELTD1 and regulating cell cycle in glioblastoma multiforme. Biochem Biophys Res Commun. 2015;467:204‐210. 26449464 10.1016/j.bbrc.2015.10.006

[42] Park I , Mukherjee J , Ito M , et al. Changes in pyruvate metabolism detected by magnetic resonance imaging are linked to DNA damage and serve as a sensor of temozolomide response in glioblastoma cells. Cancer Res. 2014;74:7115‐7124. 25320009 10.1158/0008-5472.CAN-14-0849 PMC4253720

[43] Ziegler J , Bastian A , Lerner M , et al. AG488 as a therapy against gliomas. Oncotarget. 2017;8:71833‐71844. 29069750 10.18632/oncotarget.18284 PMC5641093

[44] Kunkel P , Ulbricht U , Bohlen P , et al. Inhibition of glioma angiogenesis and growth in vivo by systemic treatment with a monoclonal antibody against vascular endothelial growth factor receptor‐2. Cancer Res. 2001;61:6624‐6628. 11559524

[45] Abdallah MG , Almugaiteeb TI , Raza MU , Battiste JD , Kim YT , Iqbal SM . Glioblastoma Multiforme heterogeneity profiling with solid‐state micropores. Biomed Microdevices. 2019;21:79. 31414186 10.1007/s10544-019-0416-7

[46] Huang J , He Y , Chen M , et al. Adenosine deaminase and adenosine kinase expression in human glioma and their correlation with gliomaassociated epilepsy. Mol Med Rep. 2015;12:6509‐6516. 26329539 10.3892/mmr.2015.4285 PMC4626129

[47] Yang X , Li D , Cheng S , et al. The correlation of bone morphogenetic protein 2 with poor prognosis in glioma patients. Tumour Biol. 2014;35:11091‐11095. 25099618 10.1007/s13277-014-2424-9

[48] Guo M , Jiang Z , Zhang X , et al. miR‐656 inhibits glioma tumorigenesis through repression of BMPR1A. Carcinogenesis. 2014;35:1698‐1706. 24480809 10.1093/carcin/bgu030

[49] Xing D , Wang J , Ou S , et al. Expression of neonatal Nav1.5 in human brain astrocytoma and its effect on proliferation, invasion and apoptosis of astrocytoma cells. Oncol Rep. 2014;31:2692‐2700. 24756536 10.3892/or.2014.3143

[50] Zeng J , Wu Y , Zhuang S , et al. Identification of the role of TRPM8 in glioblastoma and its effect on proliferation, apoptosis and invasion of the U251 human glioblastoma cell line. Oncol Rep. 2019 10.3892/or.2019.7260 31524272

[51] Iwadate Y , Matsutani T , Hirono S , Shinozaki N , Saeki N . Transforming growth factor‐beta and stem cell markers are highly expressed around necrotic areas in glioblastoma. J Neurooncol. 2016;129:101‐107. 27193555 10.1007/s11060-016-2145-6

[52] Brescia P , Ortensi B , Fornasari L , Levi D , Broggi G , Pelicci G . CD133 is essential for glioblastoma stem cell maintenance. Stem Cells. 2013;31:857‐869. 23307586 10.1002/stem.1317

[53] Izumoto S , Ohnishi T , Arita N , Hiraga S , Taki T , Hayakawa T . Gene expression of neural cell adhesion molecule L1 in malignant gliomas and biological significance of L1 in glioma invasion. Cancer Res. 1996;56:1440‐1444. 8640837

[54] Bao S , Wu Q , Li Z , et al. Targeting cancer stem cells through L1CAM suppresses glioma growth. Cancer Res. 2008;68:6043‐6048. 18676824 10.1158/0008-5472.CAN-08-1079 PMC2739001

[55] Jin X , Jin X , Jung JE , Beck S , Kim H . Cell surface Nestin is a biomarker for glioma stem cells. Biochem Biophys Res Commun. 2013;433:496‐501. 23524267 10.1016/j.bbrc.2013.03.021

[56] Wang J , Sakariassen PO , Tsinkalovsky O , et al. CD133 negative glioma cells form tumors in nude rats and give rise to CD133 positive cells. Int J Cancer. 2008;122:761‐768. 17955491 10.1002/ijc.23130

[57] Ishiwata T , Teduka K , Yamamoto T , Kawahara K , Matsuda Y , Naito Z . Neuroepithelial stem cell marker nestin regulates the migration, invasion and growth of human gliomas. Oncol Rep. 2011;26:91‐99. 21503585 10.3892/or.2011.1267

[58] Matsuda Y , Naito Z , Kawahara K , Nakazawa N , Korc M , Ishiwata T . Nestin is a novel target for suppressing pancreatic cancer cell migration, invasion and metastasis. Cancer Biol Ther. 2011;11:512‐523. 21258211 10.4161/cbt.11.5.14673 PMC3230315

[59] Wei LC , Shi M , Cao R , Chen LW , Chan YS . Nestin small interfering RNA (siRNA) reduces cell growth in cultured astrocytoma cells. Brain Res. 2008;1196:103‐112. 18234160 10.1016/j.brainres.2007.11.026

[60] Wren JD , Bekeredjian R , Stewart JA , Shohet RV , Garner HR . Knowledge discovery by automated identification and ranking of implicit relationships. Bioinformatics. 2004;20:389‐398. 14960466 10.1093/bioinformatics/btg421

